# Estimation of actomyosin active force maintained by tropomyosin and troponin complex under vertical forces in the *in vitro* motility assay system

**DOI:** 10.1371/journal.pone.0192558

**Published:** 2018-02-08

**Authors:** Shuya Ishii, Masataka Kawai, Shin'ichi Ishiwata, Madoka Suzuki

**Affiliations:** 1 Department of Physics, Faculty of Science and Engineering, Waseda University, Tokyo, Japan; 2 Department of Anatomy and Cell Biology, College of Medicine, University of Iowa, Iowa City, IA, United States of America; 3 PRESTO, Japan Science and Technology Agency (JST), Saitama, Kawaguchi, Japan; 4 Research Institute for Science and Engineering, Waseda University, Tokyo, Japan; Fachhochschule Lubeck, GERMANY

## Abstract

The interaction between actin filaments and myosin molecular motors is a power source of a variety of cellular functions including cell division, cell motility, and muscular contraction. *In vitro* motility assay examines actin filaments interacting with myosin molecules that are adhered to a substrate (e.g., glass surface). This assay has been the standard method of studying the molecular mechanisms of contraction under an optical microscope. While the force generation has been measured through an optically trapped bead to which an actin filament is attached, a force vector vertical to the glass surface has been largely ignored with the *in vitro* motility assay. The vertical vector is created by the gap (distance) between the trapped bead and the glass surface. In this report, we propose a method to estimate the angle between the actin filament and the glass surface by optically determining the gap size. This determination requires a motorized stage in a standard epi-fluorescence microscope equipped with optical tweezers. This facile method is applied to force measurements using both pure actin filaments, and thin filaments reconstituted from actin, tropomyosin and troponin. We find that the angle-corrected force per unit filament length in the active condition (pCa = 5.0) decreases as the angle between the filament and the glass surface increases; i.e. as the force in the vertical direction increases. At the same time, we demonstrate that the force on reconstituted thin filaments is approximately 1.5 times larger than that on pure actin filaments. The range of angles we tested was between 11° and 36° with the estimated measurement error less than 6°. These results suggest the ability of cytoplasmic tropomyosin isoforms maintaining actomyosin active force to stabilize cytoskeletal architecture.

## Introduction

Force produced by actomyosin interaction is essential in a wide variety of cellular functions [1,2], hence the organization of actin filament and myosin is diverse. While the contractile system is stable and regularly aligned in muscle cells, continuous modulation in structures and functions is essential in cell migration, cell division and tissue morphogenesis in non-muscle cells. *In vitro* motility assay has been a powerful experimental system to study the actomyosin interaction. This assay reconstitutes the actin and myosin interaction on a substrate (typically a glass surface) under an optical microscope by using purified contractile proteins [3–5]. It is often combined with additional techniques to observe interactions at the single molecule level. Optical tweezers are a prominent example being used among these techniques. With optical tweezers, microscopic particles such as polystyrene beads and bacteria (size can range from 20 nm to tens of μm) can be handled in a non-invasive manner and the developed force can be quantified up to tens of pN [6–9]. Optical tweezers have been successfully used to characterize molecular motors [10–17]. In particular, the *in vitro* motility assay utilizing optical tweezers has been successful in revealing the properties of actomyosin interaction: force generation and consequent motile mechanisms [10,13,14,18,19], and intra- and inter-molecular cooperativity [17,20,21]. However, in the past the force measurement was carried out only in a two-dimensional plane. Recently, Pollari and Milstein reported a method to measure vertical force by using optical tweezers, and to correct the vertical component of the trap stiffness that is affected by aberrations and interferences of laser light [22,23]. In fact, measuring the force of actomyosin interaction in three-dimensional space is essential to characterize under the various cellular conditions in which the contractile system is placed.

**Fig 1:**
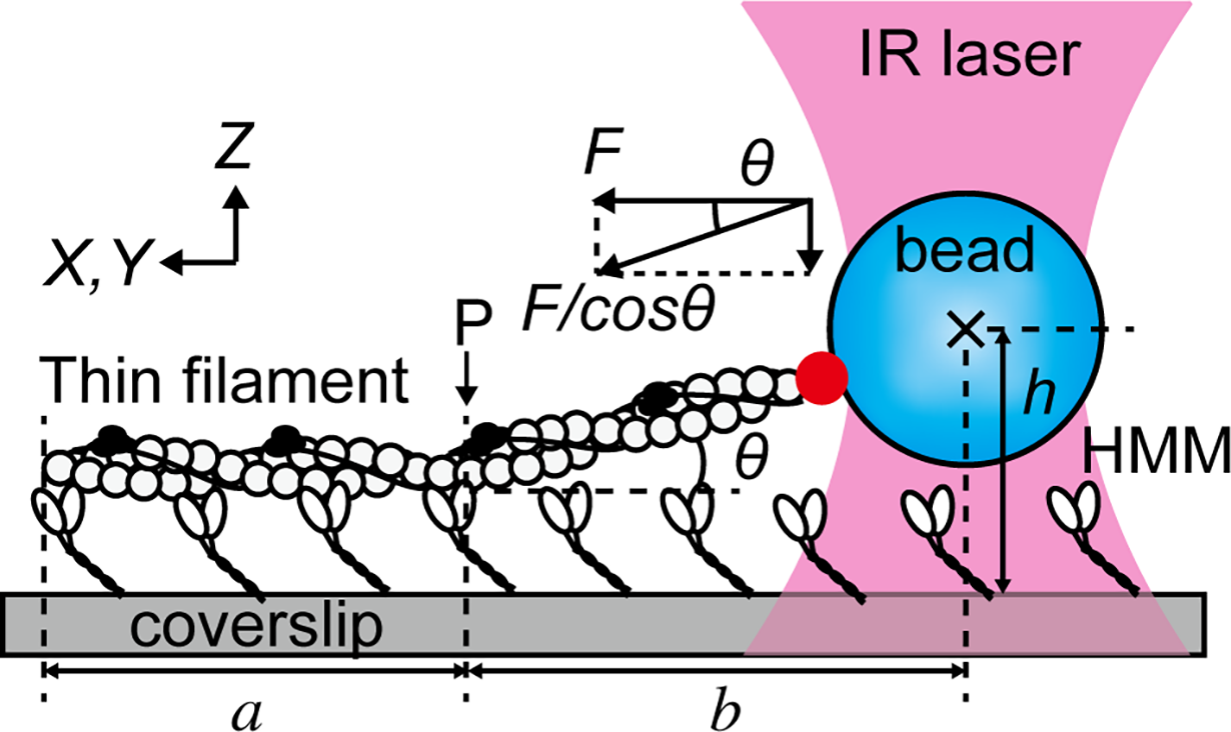
Schematic illustration of the *in vitro* motility assay system showing the parameters considered. A reconstituted thin filament (actin is illustrated by open circles, Tpm thin curved lines, and Tn black spheres) attached to a polystyrene bead (1.0 µm in diameter) via gelsolin (red sphere) was manipulated by optical tweezers. In the presence of ATP, the thin filament was interacted with HMM molecules that were attached to the surface of a collodion-coated glass coverslip. The region indicated by *a*, the part of the thin filament which interacted with HMM molecules that was visualized with the total internal reflection fluorescence (TIRF) microscopy system. The region indicated by *b*, the distance between the center of the bead and the end of the thin filament interacting with HMM (*P*), was determined from the epifluorescence microscopy. *θ*, the angle between the thin filament and the glass surface at *P*, determined geometrically from the distance of the bead from the glass surface (*h*) and *b* as *θ* = *arctan* (*h*/*b*). The actual size of HMM is small enough to be ignored compared to *h* (see text). Sliding force in horizontal direction (*F*) was obtained from the trap stiffness (range: 0.042 pN/nm to 0.15 pN/nm) and the displacement of the bead in the *X*-*Y* plane. The corrected force vector was calculated as *F*/cos*θ*.

In this report, we assessed the effects of the vertical component of force in the *in vitro* motility assay system. Individual actin filaments, or thin filaments reconstituted from actin, tropomyosin (Tpm), and troponin (Tn) were attached to a bead that was optically trapped (Fig 1). The distance between the center of the trapped bead and the coverslip (*h* in Fig 1) was determined based on the relationship between the bead image, the distance of the bead from the glass surface, and the mechanical adjustment of the optical components. The angle of the force vector (*θ*) and the dependence of force on *θ* were obtained. We found that active force (*F*/cos*θ* in Fig 1) decreases as *θ* increases, and Tpm and Tn elevates the active force in the range of *θ* studied.

## Materials and methods

### Purification of proteins

Actin and heavy meromyosin (HMM; α-chymotrypsin proteolytic cleavage of full-length myosin II) were purified from rabbit white skeletal muscle [24,25]. Two rabbits were purchased from Japan Laboratory Animals, Inc., and used for the present report. All experimental procedures conformed to the “Guidelines for Proper Conduct of Animal Experiments” approved by the Science Council of Japan and were performed according to the “Regulations for Animal Experimentation” at Waseda University; Gelsolin from bovine plasma and bovine ventricular Tn (complex of TnI, TnC and TnT) were purified as described previously [26,27]. Bovine samples were obtained as food by-products. The α-Tpm WT was provided from Chase laboratory at The Florida State University [28].

### Solutions

F-buffer contained 2 mM MgCl_2_, 1.5 mM NaN_3_, 100 mM KCl, 1 mM dithiothreitol (DTT), and 2 mM 3-(*N*-morpholino)propane sulfonic acid (MOPS), pH adjusted to 7.0. Rigor solution contained 4 mM MgCl_2_, 1 mM ethylene glycol bis (β-aminoethyl ether) N, N′-tetraacetic acid (EGTA), 25 mM KCl, 10 mM DTT, and 25 mM imidazole-HCl (Im-HCl), pH adjusted to 7.4. Relaxing solution contained 2 mM Na_2_ATP (Roche Diagnostics, Indianapolis, IN, USA), 4 mM MgCl_2_, 1 mM EGTA, 13 mM KCl, 6 mM KH_2_PO_4_, 10 mM DTT, 25 mM Im-HCl, and 1 mg/ml bovine serum albumin (BSA: Sigma-Aldrich, St. Louis, MO, USA). Activating solution contained 1 mM CaEGTA (pCa = 5.0, where pCa = –log_10_[Ca^2+^]) in place of 1 mM EGTA of the relaxing solution. pCa 9.0 solution was the mixure of relaxing and activating solutions in 99:1 ratio. Activating and pCa 9.0 solutions contained 25 mM glucose, 0.22 mg/ml glucose oxydase, and 0.036 mg/ml catalase to remove O_2_ to minimize photobleaching of fluorescent dye. pH of all solutions was adjusted to 7.4, except for F-buffer. All the measurements were carried out at 24 ± 1°C. All chemicals were purchased from Wako Pure Chemical Industries (Osaka, Japan), unless otherwise stated.

### Preparation of bead-tailed actin and thin filaments

Bead-tailed actin filaments were prepared as previously described [24,29,30]. Briefly, polystyrene beads (1.0 μm in diameter, Blue-Fluorescent, Molecular Probes, Eugene, OR, USA) were coated with a mixture of gelsolin and TMR-maleimide- (Molecular Probes) conjugated and unlabeled BSA using the carboxyl group at the surface of the beads. Polymerized actin filaments fluorescently labeled by rhodamine-conjugated phalloidin (Molecular probes) were attached to polystyrene beads through gelsolin which serves as an anchor at the barbed end of the actin filament ('bead-tailed actin filament'; 1.2 µM actin) (Fig 1). Thin filaments were reconstituted in a test tube in the presence of bead-tailed actin filaments (0.6 µM actin), 0.6 µM Tpm, and 0.6 µM Tn in F-buffer (20 µL). The mixture was incubated for 1 h on ice [31], followed by the 'annealing' treatment (45°C, 10 min) to stabilize the proper head-to-tail assemblies of Tpm dimers on an actin filament [32]. The samples were stored on ice until used for experiments.

### Flow cells

Glass coverslips (24 X 60 mm, Matsunami Glass, Osaka, Japan) were cleaned by sonication sequentially in KOH (450 mM), acetone, and 100% ethanol for 15 min each. The coverslips were rinsed with distilled water after each step. The cleaned coverslips were dried at 50°C for overnight and stored at room temperature (RT = 24°C). One day before the experiment, the surface of the coverslip was coated with collodion (0.1% dissolved in 3-methylbutyl acetate). The coverslips were dried at RT for about 10 minutes. Further incubation at 50°C overnight improved the reproducibility of the data. The coverslips were stored in a dry cabinet. A smaller coverslip (18 X 18 mm, Matsunami Glass) was glued to the larger coverslip at two opposing sides by using double-stick tape 11 mm apart, resulting in a flow-cell with a volume of ~18 µL.

### Optical setup

**Fig 2:**
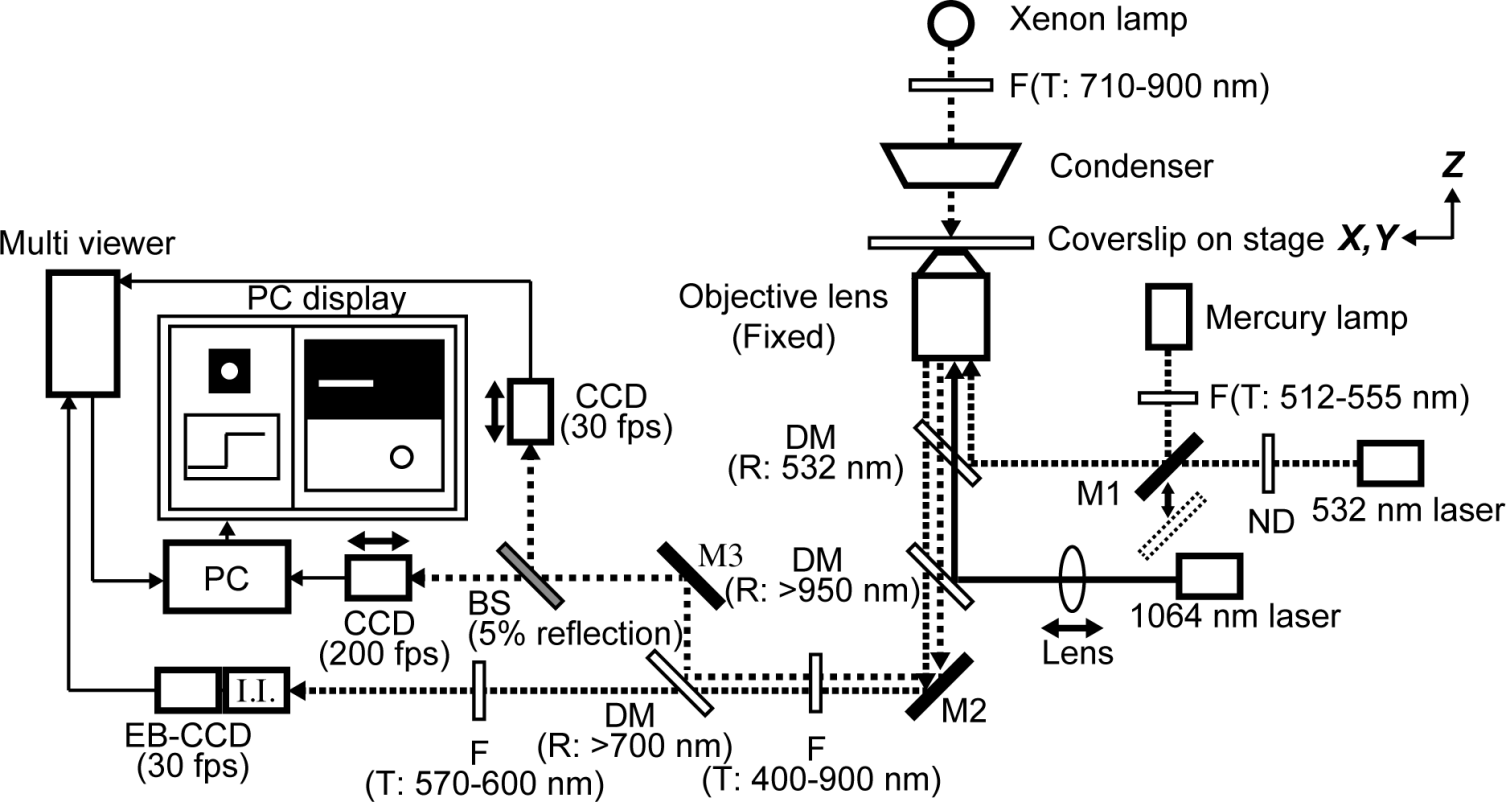
Schematic diagram of the optical setup. The optical setup for optical tweezers, bright-field microscopy and fluorescence microscopy were built on an inverted microscope. The position of the objective lens was fixed, whereas *X*, *Y* and *Z* positions of the sample stage were adjustable (indicated by arrows). The focal plane of 1064 nm laser for optical tweezers can be adjusted by shifting the position of a lens ('Lens' with a double-headed arrow placed in the light path of 1064 nm laser) along the light path. A xenon lamp was used as the light source at 710–900 nm to obtain bright-field images of beads. The bright-field images split by a beam sampler (BS) were projected onto two individual CCD cameras. One CCD camera worked at 200 fps, and its images were captured by a PC and analyzed in real time to track the position of the bead. The other CCD camera (30 fps) was connected to a multi viewer. The focal planes of these two CCD cameras were adjusted individually by the position of the camera along the light path (indicated by double-headed arrows). For fluorescence imaging, 532 nm laser and mercury lamp were used as light sources for total internal reflection microscopy and epifluorescence microscopy, respectively. These two fluorescence imaging methods could be alternated by shifting the mirror position (M1, shown with a double-headed arrow). Fluorescence images were captured by an EB-CCD camera (30 fps) serially connected with an image intensifier (I.I.) and transmitted to the multi viewer. Bright-field and fluorescence images combined by the multi viewer were shown side-by-side and captured by a PC for recording and analysis purposes. All images were shown in a PC display during the experiment. F, filter. M, mirror. DM, dichroic mirror. BS, beam sampler. ND, neutral density filter. T, transmitted wavelength. R, reflected wavelength.

The experimental apparatus was placed on a pneumatic isolation table (HA-189LY, HERZ, Kanagawa, Japan). The optical setup (Fig 2) was based on an inverted optical microscope (IX71, Olympus, Tokyo, Japan). While the position of the objective lens (Apo TIRF 100X oil-immersion, N.A. = 1.49, Nikon, Tokyo, Japan) was fixed, the sample stage was controlled by a three-axis stepping motor (MP-285, Sutter Instrument Co., Novato, CA, USA). Nd-YAG laser beam (T20-BL-106C, 1064 nm, 1W, Spectra-Physics, Santa Clara, CA, USA) was focused on the sample stage by the objective lens to trap a polystyrene bead. For epifluorescence and total internal reflection fluorescence (TIRF) microscopies, a mercury lamp (BH2-RFL-T3, filtered to 512–555 nm, Olympus) and a green laser (532 nm, Melles Griot KK, Tokigawa, Saitama, Japan), respectively, were reflected by a dichroic mirror (reflecting 532 nm) positioned just behind the objective lens. These excitation light sources were alternately used depending on an illumination purpose. A Xenon lamp (MAX-303, filtered to 710–900 nm, Asahi Spectra, Tokyo, Japan) was used as a light source for bright-field microscopy. Fluorescence and bright-field images were collected at 400–900 nm by a band-pass filter, and then separated by a dichroic mirror (reflected wave length, R > 700 nm). Fluorescence images were further filtered to 570–600 nm, amplified by an image intensifier (Video Scope international, Ltd., Dulles, VA, USA), and recorded using an electron bombardment CCD camera (EB-CCD, MC681SPD-ROBO, Texas Instruments, Inc., Dallas, TX, USA). Bright-field images were split by using a beam sampler (~5% reflection, OptoSigma Co., Ltd., Hidaka, Saitama, Japan) and monitored by two individual CCD cameras. One camera (MC-781P; Texas Instruments) was operated at a video rate (30 fps), and the other high speed camera (IMPERX, Inc., Boca Raton, FL, USA) was operated at 200 fps. Bright-field and fluorescence images, both monitored at 30 fps, were combined using a multi-viewer (MV-40F, FOR-A, Tokyo, Japan) and stored in a Windows PC via a video capture board (The Imaging Source LLC, Charlotte, NC, USA) with a custom-build program based on LabVIEW (National Instruments Japan, Tokyo, Japan). Fluorescence intensity was monitored in real time using LabVIEW program to adjust the stage position to focal plane of fluorescence imaging. Recorded images were later analyzed by ImageJ software with another PC (Apple Japan, Tokyo, Japan). Displacement of the bead was determined by a plug in in ImageJ software (Particle Track and Analysis). The position of the bead was determined by fitting the 2D distribution of pixel values to 2D Gaussian distribution. Images of the high speed camera were captured by using another video capture board (NI PCIe-1430, National Instruments Japan) and the displacement of the bead was tracked in real time by LabVIEW program and displayed on a PC screen for ease of measurement. In this analysis, each image was binarized, and the center of the bead was determined as the center of a circle approximating the bead. Relative positions of the focal planes of fluorescence and bright-field microscopies and the center of the optical trap were adjusted independently by the positions of lenses and cameras (Fig 2). Equipment and the air conditioner of the room were turned on at least one hour earlier than measurements to control the experimental temperature to 24 ± 1°C.

### Procedure to determine the height of the trap center from the focal plane of the fluorescence microscopy

**Fig 3:**
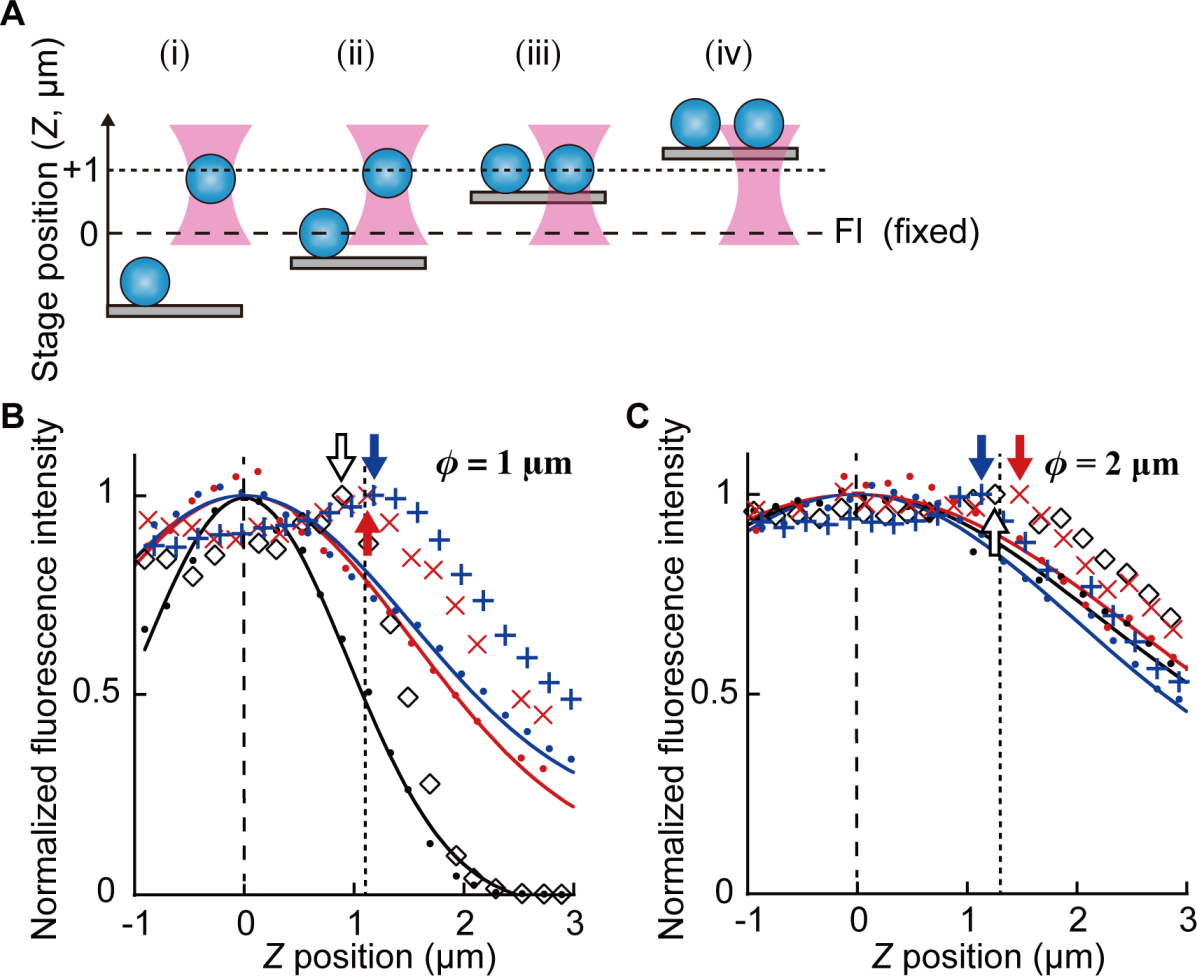
The procedure to determine the height of the trap center from the focal plane of the fluorescence microscopy. (A) Schematics showing the procedure to measure the trap height. Objective lens is fixed in space. (i) The focal plane of the bright-field microscopy (dotted line, adjustable by the position of the camera) is preset at about 1 μm above that of the fluorescence microscopy (dashed line). A floating bead is trapped. (ii) While the other bead is attached to the coverslip, the sample stage is elevated in steps (200 nm each). (iii) When the coverslip touches the trapped bead, the bead is adsorbed to the coverslip. (iv) Both beads are elevated together with the sample stage. (B and C) Data showing the changes in fluorescence intensity during the trap height measurement obtained with beads with the diameter of 1.0 μm (B) or 2.0 μm (C). Dots represent the change in the fluorescence intensity of the beads adsorbed to the coverslip when the sample stage was lifted upwards. The symbols (+, x, ◊) indicate the fluorescence intensity of the trapped beads. Three individual trials are represented by three different colors. The fluorescence intensities were determined in square regions. The size for the beads with the diameter of 1.0 μm was 2.2 μm X 2.2 μm for the data indicated in black or 1.8 μm X 1.8 μm for the data indicated in blue and red. For the beads with the diameter of 2.0 μm, the size was 3.3 μm X 3.4 μm. The change of the fluorescence intensities along the *Z* axis can be fit to the Gaussian distribution (shown in thin curves with the same color as corresponding dots). The peak position of the Gaussian distribution is the focal plane of the fluorescence microscopy (*Z* = 0, vertical dotted line). After the intensity peaks at about *Z* = 1 μm (arrows with the same color as corresponding symbols), it begins to decrease in the same way as those of the bead adsorbed to the coverslip. This is because the trapped bead is adsorbed to the coverslip and it moves together with the pre-adsorbed bead. From the peak positions (1.1 ± 0.15 μm and 1.3 ± 0.18 μm for beads with the diameter of 1.0 μm and 2.0 μm, respectively; mean ± SD, vertical dashed line), we estimated the distance of the trapped 1.0 μm beads from the focal plane as *Z* = 0.88 μm after the correction for aberration (see main text for details).

Fluorescent polystyrene beads (1.0 or 2.0 μm in diameter, yellow-green, Molecular Probes) dispersed in rigor buffer (1000-fold dilution) were infused into the flow cell. After sealing two open ends of the flow cell with non-fluorescent nail polish, the flow cell was placed on the microscope stage. Epifluorescence microscopy was used in this measurement. The sample stage was elevated in 200 nm steps, and a microscopic image was recorded at each step. The images were averaged for five seconds (150 frames). The fluorescence intensity of the bead was determined as the average of pixel values in a square region fixed over the bead. The square regions were set to 2.2 μm X 2.2 μm or 1.8 μm X 1.8 μm for the beads with the diameter of 1.0 μm, and 3.3 μm X 3.4 μm for the beads with the diameter of 2.0 μm (cf. Fig 3).

### Calibration of the trap stiffness of the optical tweezers

The stage was moved to one direction at the velocity *v* = 500 μm/s to measure the displacement of the trapped bead. The viscous force was calculated based on viscous resistance *F*_*vis*_ [33].

$$F_{vis}=\frac{6\pi \eta rv}{1-\frac{9}{32}(\frac{r}{h})+\frac{1}{64}{\left(\frac{r}{h}\right)}^{3}-\frac{45}{4096}{\left(\frac{r}{h}\right)}^{4}-\frac{1}{512}{\left(\frac{r}{h}\right)}^{5}}$$ (1),

where *η* = 0.89 mPa·s is the viscosity of water at 25°C [34], *r =* 1.0 μm is the diameter of the bead, and *h* is the distance between the center of the trapped bead and the surface of coverslip. The calibration was carried out at the same height as in the sliding force measurement to avoid any contribution of spherical aberration on trap stiffness that depends on the distance from the coverslip (discussed later) [22,23,35–37]. The trap stiffness was adjusted to 0.042–0.15 pN/nm by regulating the laser power, so that the displacement of the bead was less than 200 nm, which is within the linear range of our optical tweezers.

### Measurement of the sliding force in *in vitro* motility assay under TIRF microscopy

**Fig 4:**
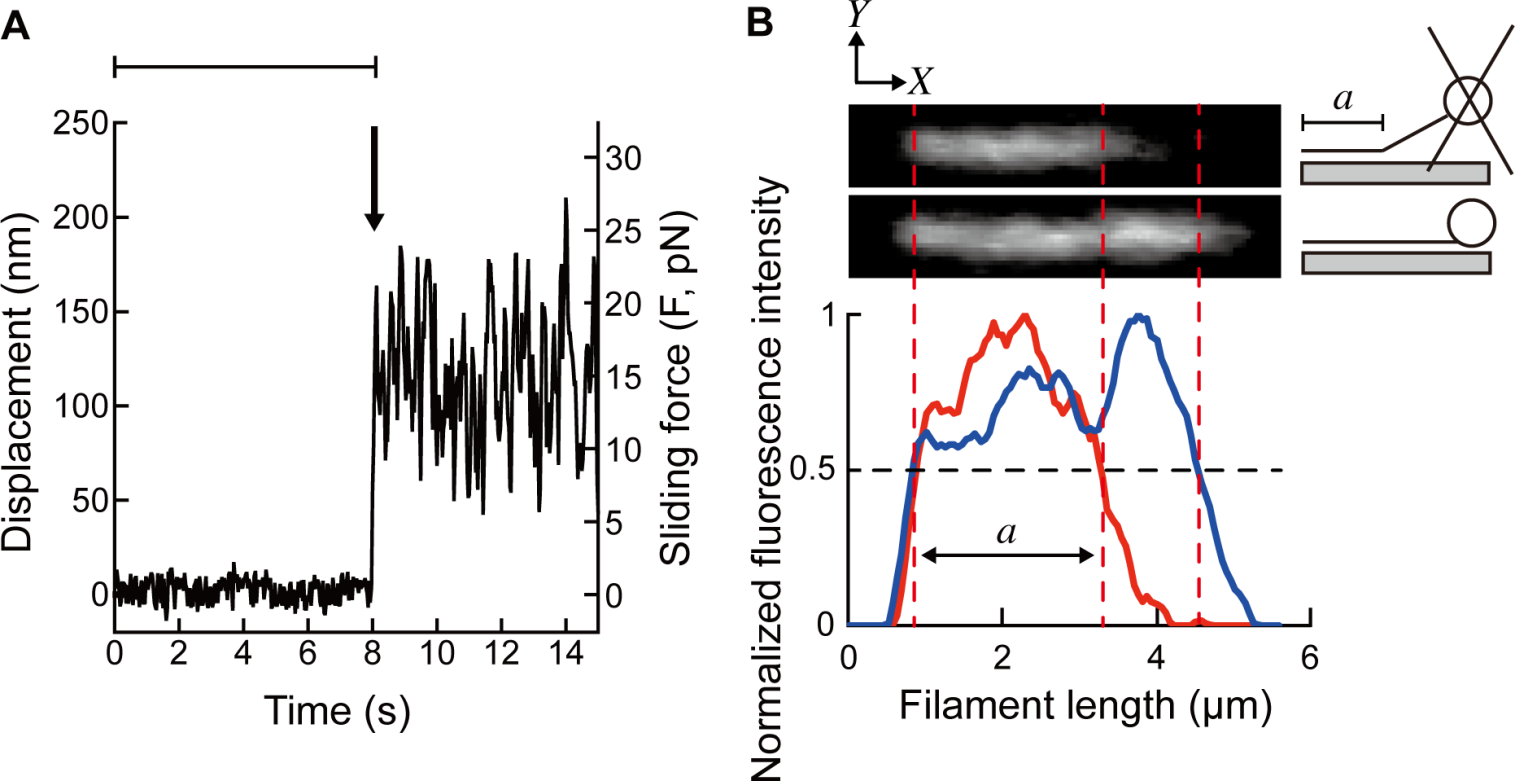
Analysis of sliding force and filament length. (A) A representative time course of the bead displacement during measurement. At first, a bead-tailed reconstituted thin filament (*a* = 3.3 μm) was trapped in the activating solution, where the displacement of the bead is defined as 0 (the bead is at the trap center) because there is no interaction between the thin filament and HMM (during the period shown by a horizontal line). Then, the coverslip was elevated to the focal plane of the fluorescence microscopy, which is below the focal plane of the bright-field microscopy and the focus of the optical tweezers. When the thin filament started to interact with HMM on the coverslip, the bead was displaced away from the trap center (arrow). To calculate the sliding force in the *X-Y* plane, the displacement was multiplied by the trap stiffness. (B) Representative fluorescence images of the same bead-tailed actin filament trapped (upper photo) and released (lower photo), and the result of their intensity analysis. While the bead is trapped above the glass surface, only a part of the actin filament interacting with HMM on the coverslip was visible under the TIRF microscopy. Therefore, the actin filament appears shorter when the bead is trapped (upper photo) than when it is released (lower photo). Fluorescence images shown are the region of interest (ROI) within which the fluorescence intensities were averaged along the columns in *Y*-axis to produce the 1D intensity profile along the *X*-axis (red and blue curves correspond to the upper and lower fluorescence micrographs, respectively). Background signal was determined from the other ROI (set next to the actin filament) which was subtracted from the signal of the actin filament. Frequently, the background was none. The parameter '*a*' as defined in Fig 1 was determined as the distance between the points where the fluorescence intensity was half of the maximum (bottom).

Measurement of the sliding force was carried out as reported [31,38,39]. From one side of the flow cell, 20 μL of HMM solution (30 μg/mL in rigor solution) was injected. After 60 sec, another 20 μL of HMM solution was injected from the other side of the flow cell. After 60 sec, the unattached HMM molecules were washed out using 20 μL of the activating solution or pCa 9.0 solution containing BSA (5 mg/ml). After 5 min, 50 μL of the experimental solution containing bead-tailed filaments was injected. For the bead-tailed thin filaments, the stored sample was diluted to 1:100 just before the injection into the flow cell; 100 nM Tpm and 100 nM Tn were present in the experimental solution to ensure that Tpm and Tn were bound on the thin filaments throughout the measurements [40]. Measurements were performed using the TIRF microscopy. The position of the trapped bead was determined by fitting the intensity of the bright-field image of the bead to a Gaussian distribution. The length of the part of the filament interacting with HMM (= *a*) was determined as the distance between the points where the fluorescence intensity was half maximum (cf. Fig 4B). *a* of 2.7 ± 0.60 and 2.8 ± 0.67 μm in reconstituted thin filament and actin filament, respectively, were obtained. The numbers of flow cell we used were 10 and 15 for the force measurements with reconstituted thin filament and actin filament, respectively. Measurements of up to 15 times per each flow cell were performed in each condition. In order to average the various densities of HMM molecules that were possibly present within and among preparations, multiple areas were used in each coverslip. All measurements were completed within 30 min to avoid a formation of rigor bonds as a result of the ATP consumption in the flow cell.

## Results and discussion

### Height of the trapped bead determined from the fluorescence microscopy

We determined the distance between the center of the trapped bead and the focal plane (*h* in Fig 1) from the fluorescence intensity of the bead (Fig 3). Under the epifluorescence microscopy with the fixed focal plane, the fluorescence intensity of the bead adsorbed to the glass coverslip was monitored as the sample stage was elevated along the *Z* axis (Fig 3A, the bead on the coverslip). The intensity approximated the Gaussian distribution (Fig 3B and 3C, solid lines). This correlation is used to locate the three-dimensional position of the bead, as reported previously [41]. Here, the *Z* position at which the Gaussian distribution had a peak value was defined as the focal plane of the fluorescence microscopy (*Z =* 0 μm). On the other hand, the fluorescence intensity of the trapped bead had a peak at around *Z =* 1 μm, and decreased as the coverslip was elevated further above the peak (Fig 3B and 3C, symbols). The oil-immersion lens used in this study is suitable for observing specimens at the surface of the coverslip. As the coverslip approached the trapped bead, the thickness of the water layer between the bead and the coverslip surface decreased to reduce the effect of spherical aberration [42], which may contribute to the increment of the fluorescence intensity before the coverslip touched the trapped bead. When the coverslip touched the bead, it was adsorbed to the coverslip and then moved together with pre-adsorbed beads, i.e. the fluorescence intensity of both beads decreased in the same way. We have tested beads with two different diameters. For the beads with the diameter of 1.0 μm (Fig 3B) and 2.0 μm (Fig 3C), the peak positions in fluorescence intensity were measured as 1.1 ± 0.15 μm and 1.3 ± 0.18 μm (mean ± SD, *n* = 3), respectively. In general, trapped bead is shifted downstream of the focus due to a scattering force of the trapping laser [43]. The difference in trap positions between beads with diameters 1.0 μm and 2.0 μm is consistent with the simulated results as previously reported; that the larger bead is balanced more downstream of the trapping laser [37,44,45]. With a similar method, Lang *et al*. determined *h* by using the signal measured by a photo diode [46]. Alternatively, *h* can be measured by back-focal-plane interferometry [23,36], or from the intensity of scattered evanescent light [47]. Relative position of a particle to the focal plane is determined in magnetic tweezers using a series of concentric circles surrounding the particle image [48]. Coherent light is used for the illumination in this measurement.

Due to the spherical aberration, the actual distance between the center of the bead and the coverslip was 0.8 times of the measured value (i.e. the bead moves away when the coverslip approaches it. When the coverslip is moved away from the bead, the bead approaches the coverslip) [23,36]. Thus, we estimated the value of *h* for the 1.0 μm bead as 0.88 μm (= 1.1 μm X 0.8). Finally, the focus of the bright-field microscopy was set at the center of the trapped bead for the subsequent force measurement.

### Measurement of the sliding force and the angle of force vector

The bead to which a single filament was attached was trapped by the optical tweezers. After the filament was aligned parallel to the coverslip by moving the sample stage in one direction on the *X*-*Y* plane, the sample stage was positioned at the focal plane of the florescence microscopy. This is below the focus of the bright-field microscopy and the optical tweezers, where the fluorescence intensity of the field of view monitored in real time was at its maximum. Then the tip of the filament was attached to the coverslip surface and the bead started to deviate from the trap center as the active force developed by the actomyosin interaction (Fig 4A). Sliding force (*F*) was determined as the average of the displacement multiplied by the trap stiffness of the optical tweezers.

**Fig 5:**
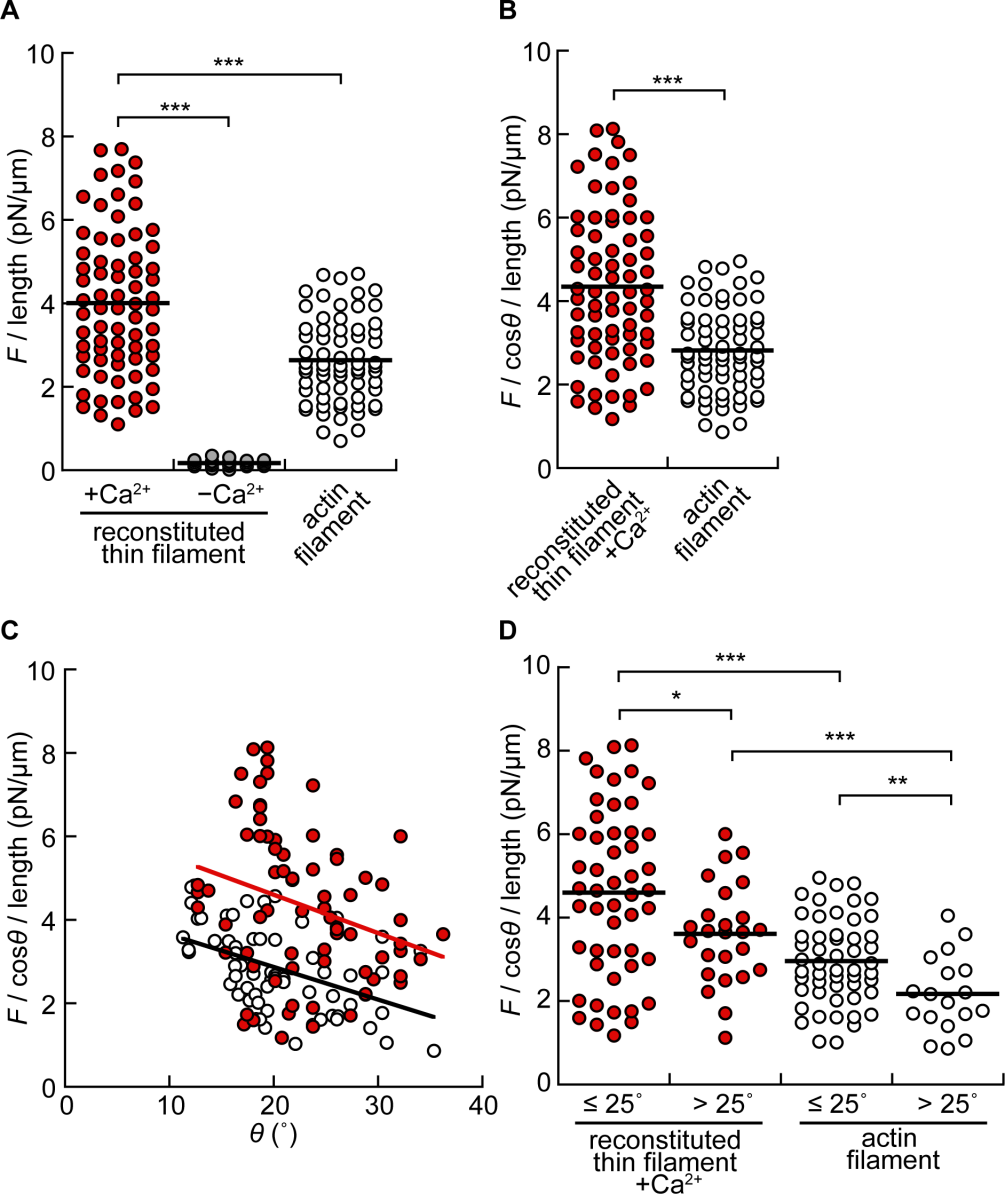
Correlation between corrected sliding force per unit length and angle of the force vector. (A) Distribution of sliding force (*F*) per unit length of filament. Reconstituted thin filament (red circles); +Ca^2+^, 4.0 ± 1.7 pN/μm (*n* = 71);–Ca^2+^, 0.17 ± 0.08 pN/μm (*n* = 22). Actin filament (white circles); 2.6 ± 1.0 pN/μm (*n* = 66). (B) Distribution of *F* per unit length of filament with a compensation of the angle of force vector (cos*θ*). Horizontal bars indicate average values. Reconstituted thin filament, 4.3 ± 1.8 pN/μm. Actin filament, 2.8 ± 1.0 pN/μm. (C) Correlation between (*F*/cos*θ*) per unit length of filament and the angle between the thin filament and the glass surface (*θ*). Regression lines (red for reconstituted thin filament, y = – 0.092x + 6.4, R = 0.28; black for actin filament, y = – 0.078x + 4.4, R = 0.45) are for visual guide. (D) Distribution of *F* per unit length of filament with a compensation of the angle of force vector (cos*θ*) at 0°< *θ* ≤ 25° or *θ* > 25°. Reconstituted thin filament; 4.6 ± 2.0 pN/μm at 0°< *θ* ≤ 25°(*n* = 48), 3.7 ± 1.1 pN/μm at *θ* > 25° (*n* = 23). Actin filament; 3.0 ± 1.0 pN/μm at 0° < *θ* ≤ 25°(*n* = 50), 2.2 ± 0.91 pN/μm at *θ* > 25° (*n* = 16). Red and gray symbols indicate data obtained from reconstituted thin filaments in the presence (*n* = 71) and in the absence (*n* = 22) of Ca^2+^, respectively. White symbols indicate data obtained from actin filaments (*n* = 66). Data were statistically compared by using two-sided student's t-test (*0.01≤ p < 0.05, **0.001≤ p < 0.01, ***p < 0.001).

Reconstitution of Tpm/Tn has been shown to enhance actomyosin force generation in the presence of Ca^2+^ [31,39,49,50]. This phenomenon was also observed with our experiments by comparing the sliding force per unit length of the filament, which was obtained by dividing force (*F*) by the length of the filament interacting with HMM (region *a*, Fig 1). Only this part of the filament was visible under TIRF microscopy during force measurements (Fig 4B). The *F*/length in the activating solution increased to 4.0 ± 1.7 pN/μm in reconstituted thin filaments (*n* = 71) from 2.6 ± 1.0 pN/μm in pure actin filaments (*n* = 66) (Fig 5A). The efficacy of reconstitution was confirmed by the active force production in the absence of Ca^2+^ (pCa 9.0) (0.17 ± 0.081 pN/μm, *n* = 22) (Fig 5A). The stable attachment of reconstituted thin filaments to HMM in the absence of Ca^2+^ in the *in vitro* motility assay system was probably caused by the electrostatic interaction as discussed previously [50].

We also have considered that the trapped bead was pulled vertically in addition to horizontally due to the gap from the coverslip surface. The angle between the filament and the glass surface, *θ*, is estimated as *θ* = arctan (*h*/*b*), where *b* is the distance between the end of the visible filament closer to the trapped bead and the center of the bead (Fig 1). The height of HMM (Fig 1) can be disregarded because it is much smaller (order of 10 nm) than *h* (order of 1 μm). Thus, the corrected force vector, *F*/cos*θ*/length, was obtained as 4.3 ± 1.8 and 2.8 ± 1.0 pN/μm in reconstituted thin filament and actin filament, respectively (Fig 5B).

### Estimated measurement errors

We evaluated the possible sources of errors in measuring the length of filaments (*b*) and the trap height (*h*), and the errors in *θ* caused by these values. To estimate the amount of possible errors in the measurement of *a*, the depth of focus was first considered. The length of the part of the thin filament interacting with HMM, *a*, was determined from the fluorescence image as the distance between the points where the fluorescence intensity is half the maximum under TIRF microscopy. Toyoshima indicated that HMM molecules that support the filament motility were bound to the glass coverslip coated with nitrocellulose (collodion) near the junction between HMM and light meromyosin [51]. Observation using electron microscopy suggested that sliding filaments are not present at the tip of myosin heads but they are embedded between the heads at the surface of mica film [52]. Based on these reports, we assumed the distance from the coverslip coated by collodion to the filament to be the sum of the size of a myosin head (19 nm) [53] and the radius of an actin filament (7/2 = 3.5 nm) [54]: 19 + 3.5 = 22.5 nm. The evanescent light at 22.5 nm was calculated to be decreased by half at 80 nm from the coverslip under the current conditions [47,55] (S1 Fig). Therefore, a part of *b* near the point *P*, the end of the filament interacting with HMM (Fig 1), could be illuminated while it was within 80 nm from the coverslip. The part of the filament emitting fluorescence within the depth of field, ± 182 nm (= ±*λ*X*n*_*3*_ /2(*N*.*A*.)^2^, where *λ* = 532 nm, *n*_*3*_ = 1.52 is the refractive index of immersion oil, and *N*.*A*. = 1.49 is the numerical aperture of the objective lens; the depth of field can be obtained based on the wave optics for objective lens with a high numerical aperture), is observed as in one same focal plane [56]. The projection of this part onto the *X*-*Y* plane, 57.5/tan*θ* (57.5 nm = 80 nm– 22.5 nm), may have increased the apparent value of *a* (cf. Fig 1). Because *θ* ranged from 11° to 36° (S2A Fig; reconstituted thin filament: 23 ± 5.6°; actin filament: 21 ± 5.9°), the value 57.5 nm/tan*θ* could vary between 48 nm and 175 nm. Even with 175 nm, it was only up to ~6.4% of the average length of *a*. Thus, we conclude that the contribution of this error is negligible for determining *a*. The thickness of an actin filament was measured as 0.57 ± 0.10 μm (*n* = 3) in TIRF microscopy (determined as the half-width of fluorescence intensity along the *Y* axis in Fig 5B), while the actual dimension was about 7 nm. The same 'thickening' effect seems to take place both at the tip of the filament and at *P*. Consequently, *a* should appear longer (*b* should be shorter) than its actual length. Therefore, we conclude that the value of *a* is overestimated by 21% (= 0.57/2.7), and, *b* is underestimated by the same amount.

The trap height, *h*, could also contain an error. *h* is considered to be variable between each measurement because the bead was pulled downwards to a different degree along the *Z* axis in addition to its displacement in the *X*-*Y* plane. The displacement of the trapped bead from the trap center in the *Z* axis was estimated by dividing the *Z* axis component of force (*F* X *tanθ*) by the trap stiffness along the *Z* axis (estimated to be one-fifth of that in the *X*-*Y* plane [57]) (*ΔZ*; 161 ± 78 nm and 147 ± 48 nm for reconstituted thin filament and actin filament, respectively; S2B Fig).

By including the contributions from all sources of errors considered (*b*' = *b* + 0.57/2 μm; *h' = h–ΔZ*), the largest error in *θ* is estimated to be about 6° (= *θ'*–*θ*, where *θ'* = arctan(*h'*/*b'*); 6.0 ± 2.3° and 5.1 ± 2.0° for reconstituted thin filament and actin filament, respectively). We believe that this value has no significant contribution in the following discussion.

### Sustained force generation on reconstituted thin filament under vertical forces

Reconstitution of Tpm/Tn not only enhanced force generation in the presence of Ca^2+^, but it also stabilized the actomyosin interaction in the presence of vertical force on the filament. The corrected force vectors were distributed broadly around the mean values in both reconstituted thin filament and actin filament (Fig 5A and 5B). As shown in S2A Fig, *θ* had a range. Therefore, we hypothesized that the broad distribution of force was caused by the range of *θ*. To test this hypothesis, the actomyosin force was further plotted against *θ*. This analysis demonstrated that the force on both filaments decreased as *θ* increased (Fig 5C and 5D). This *θ*-dependence of generated force was always present in each preparation (S3 Fig). Furthermore, the force on reconstituted thin filament was about 1.5 times larger than that on actin filament at both 0°< *θ* ≤ 25° and *θ* > 25° (Fig 5D). The data in reconstituted thin filaments exhibited a larger scatter than that in pure actin filaments. It may be thought that a part of the Tpm/Tn complex is detached from the reconstituted thin filaments, resulting in less force generation. However, this is not the case because we have demonstrated the efficacy of reconstitution in the absence of Ca^2+^ (pCa 9.0) in which no force was generated (Fig 5A). The scatter of the data may reflect a variation in the density of HMM molecules in different coverslips. Areas used for the measurements were changed every time to randomly sample the preparation (see Materials and methods).

The vertical force imposed on each actomyosin interaction may be the main reason for the decrease in the active sliding force with increasing *θ* (Fig 5C and 5D). Isambert *et al*. reported the persistent lengths of reconstituted thin filament and actin filament as 12 μm and 9 μm, respectively, in the presence of Ca^2+^ [58]. Fujime and Ishiwata also reported similar data obtained by quasi-elastic scattering of laser light [59,60]. The larger this parameter is, the more rigid the polymer is. In the current study, the filaments used for measurements (reconstituted thin filament, 4.6 ± 1.1 μm; actin filament, 5.0 ± 1.2 μm) were comparable to or shorter than their persistent lengths. Therefore, we can consider that the trapping force vertical to the *X*-*Y* plane is the same throughout the filament.

The reason why the force generation on reconstituted thin filament is larger than that on pure actin filament may be attributed to more stable binding of HMM to the thin filament than to the actin filament. In fact, we previously showed that the unbinding force of actomyosin rigor bond is about 1.2 times larger in reconstituted thin filament than in the actin filament [30]. The reconstitution recovers the kinetic constants of each elementary step of the cross-bridge cycle, as demonstrated in muscle fibers by using the thin filament-extraction and reconstitution method [49,61]. The enhanced stability of actomyosin interaction by Tpm/Tn complex may contribute to the sustainable force generation regardless of *θ*, even when the force along the *Z* axis was larger on reconstituted thin filament (4.4 ± 2.0 pN, S2C Fig) than that on actin filament (2.7 ± 0.84 pN).

Takagi *et al*. have demonstrated the mechanosensing property of the myosin molecule in that the force generation of actomyosin can vary depending on the external force imposed on the actin filament [62]. We infer from our results that the Tpm/Tn complex enhances the stability of actomyosin interaction against the pulling force in the direction perpendicular to the long axis of the filament. In the cytosol of non-muscle cells, cytoskeletal isoforms of Tpm are known to regulate the interactions between the actin filament and actin binding proteins including myosin [63–66]. While the regulatory mechanisms of Tpm have been intensively studied as a part of contractile function, Hundt *et al*. have suggested that cytoplasmic Tpm plays an important role in cytoskeletal rearrangements by enhancing the processivity of myosin in the presence of external force [65]. Their results suggest that detailed measurement of the three-dimensional force generation by actomyosin in the presence of actin regulatory proteins would be one important target. Rigid and stable three-dimensional lattice structure as it exists in muscle cells is absent in the cytosol of non-muscle cells. By switching the sustainability on and off in force production, regulatory proteins such as Tpm may play an important role to adapt the mechanochemistry of actomyosin assembly, and to flexibly manage cellular functions.

## Supporting information

S1 FigIntensity of evanescent light depends on the distance from the surface of the coverslip.Calculated relative intensity of evanescent light as a function of the distance from the surafce of the coverslip with differenct incident angles of the laser beam. Red; 60.4° which is the critical angle for total reflection on coverslip (*θ*_*c*_ = sin^-1^(*n*_*2*_/*n*_*1*_) = 60.4°, where *n*_*1*_ = 1.53 is the refractive index of glass, and *n*_*2*_ = 1.33 is the refractive index of water). Orange; 64°. Green; 68.6° which is the angle set in the current study. The broken line points the relative intensity at 22.5 nm from the coverslip, representing the location of the filament during measurements. The distance where the intensity of the evanescent light is decreased by half from that at 22.5 nm is indicated by the dotted line. Blue; 72°. Purple; 76.9° which is the maximum angle to cause evanescent light (*θ*_*max*_ = sin^-1^(N.A./*n*_*1*_), where N.A. = 1.49 is the numerical aperture of the objective lens). The intensity of the evanescent light [*E*(*z*)] decreases as a function of the distance from the boundary (*z*) [49][57] as; $E(z)=E_{0}e^{-\beta z}$ (2), where $\beta =\frac{4\pi }{\lambda }\sqrt{n_{1}^{2}{sin}^{2}\theta_{i}-n_{2}^{2}}$ (3) *E*_*0*_, intensity at the boundary. *λ* = 532 nm, wavelength of the laser light.(TIF)

S2 Fig Distributions of *θ*, displacement of the trapped bead in the *Z* axis, and vertical component of the force vector. (A) Distributions of *θ*. Reconstituted thin filament, 23 ± 5.6°. Actin filament, 21 ± 5.9°. (B) Displacement of the bead in the *Z* axis calculated from *F*, *θ* and the trap stiffness in the *Z* axis that was assumed to be one-fifth of that in *X-Y* plane as previously reported [59]. Reconstituted thin filament; 161 ± 78 nm. Actin filament; 147 ± 48 nm. (C) Distribution of the perpendicular component of sliding force with a compensation of the angle of force vector (*F*/cos*θ*). Reconstituted thin filament; 4.4 ± 2.0 pN/μm. Actin filament; 2.7 ± 0.84 pN/μm. Red and white symbols represent data obtained from reconstituted thin filaments (*n* = 71) and actin filaments (*n* = 66), respectively. Horizontal bars indicate average values.
(TIF)

S3 Fig Variety in the correlations between *F* per unit length of filaments and *θ* among coverslip preparations. Data were reproduced from Fig 5C for reconstituted thin filaments (A) and actin filaments (B). Plots with the same style and color were obtained from the same preparation. The number of flow cells is 10 (A) and 15 (B).
(TIF)
