# Supplementary figures and images for: Estimation of actomyosin active force maintained by tropomyosin and troponin complex under vertical forces in the in vitro motility assay system
Source: PLoS One. 2018 Feb 8;13(2):e0192558. doi: 10.1371/journal.pone.0192558 (PMC5805308; doi:10.1371/journal.pone.0192558)

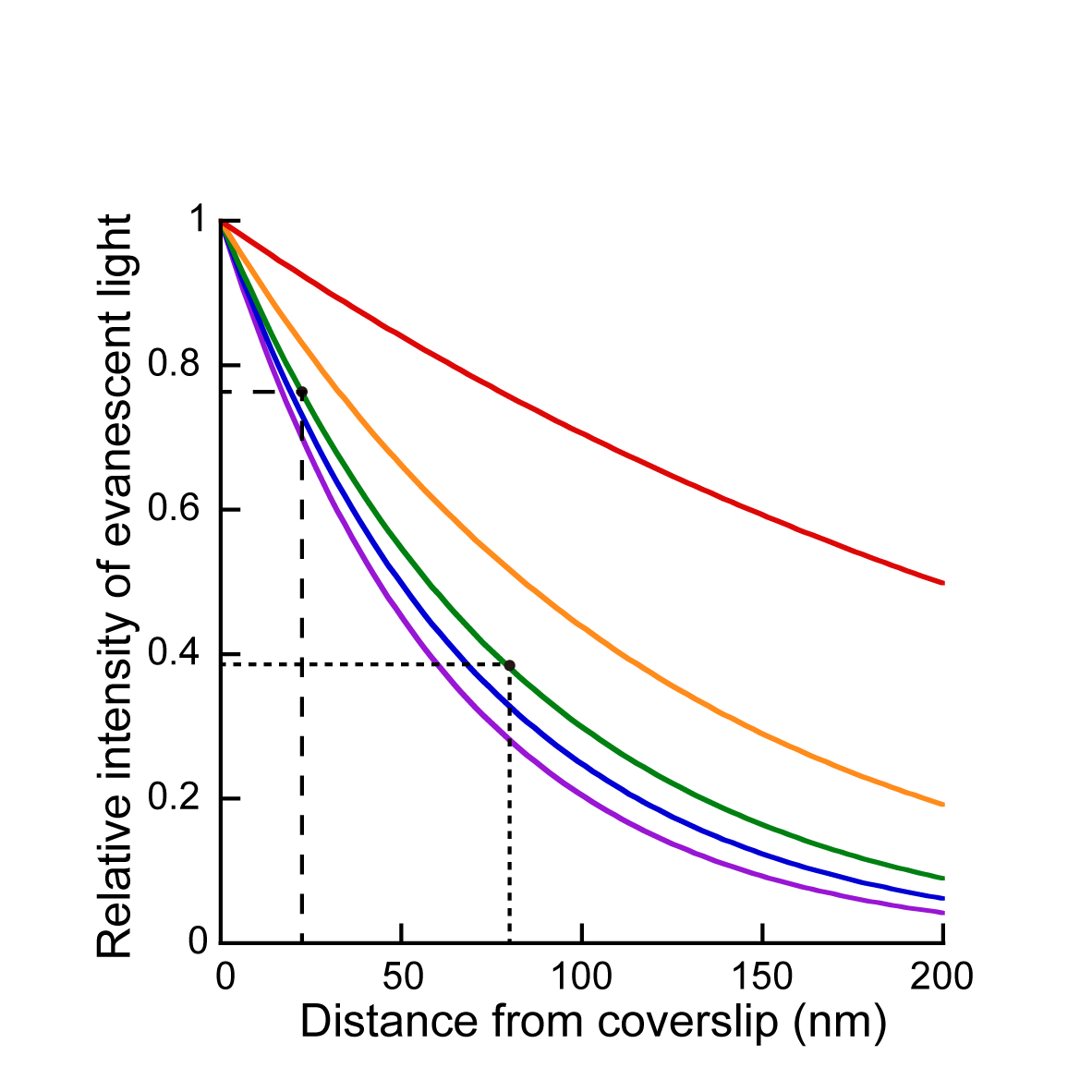

Supplement: S1 Fig — Calculated relative intensity of evanescent light as a function of the distance from the surafce of the coverslip with differenct incident angles of the laser beam. Red; 60.4° which is the critical angle for total reflection on coverslip (θc = sin-1(n2/n1) = 60.4°, where n1 = 1.53 is the refractive index of glass, and n2 = 1.33 is the refractive index of water). Orange; 64°. Green; 68.6° which is the angle set in the current study. The broken line points the relative intensity at 22.5 nm from the coverslip, representing the location of the filament during measurements. The distance where the intensity of the evanescent light is decreased by half from that at 22.5 nm is indicated by the dotted line. Blue; 72°. Purple; 76.9° which is the maximum angle to cause evanescent light (θmax = sin-1(N.A./n1), where N.A. = 1.49 is the numerical aperture of the objective lens). The intensity of the evanescent light [E(z)] decreases as a function of the distance from the boundary (z) [49][57] as; E(z)=E0e−βz (2), where β=4πλn12sin2θi−n22 (3) E0, intensity at the boundary. λ = 532 nm, wavelength of the laser light. (TIF) [file pone.0192558.s001.tif]

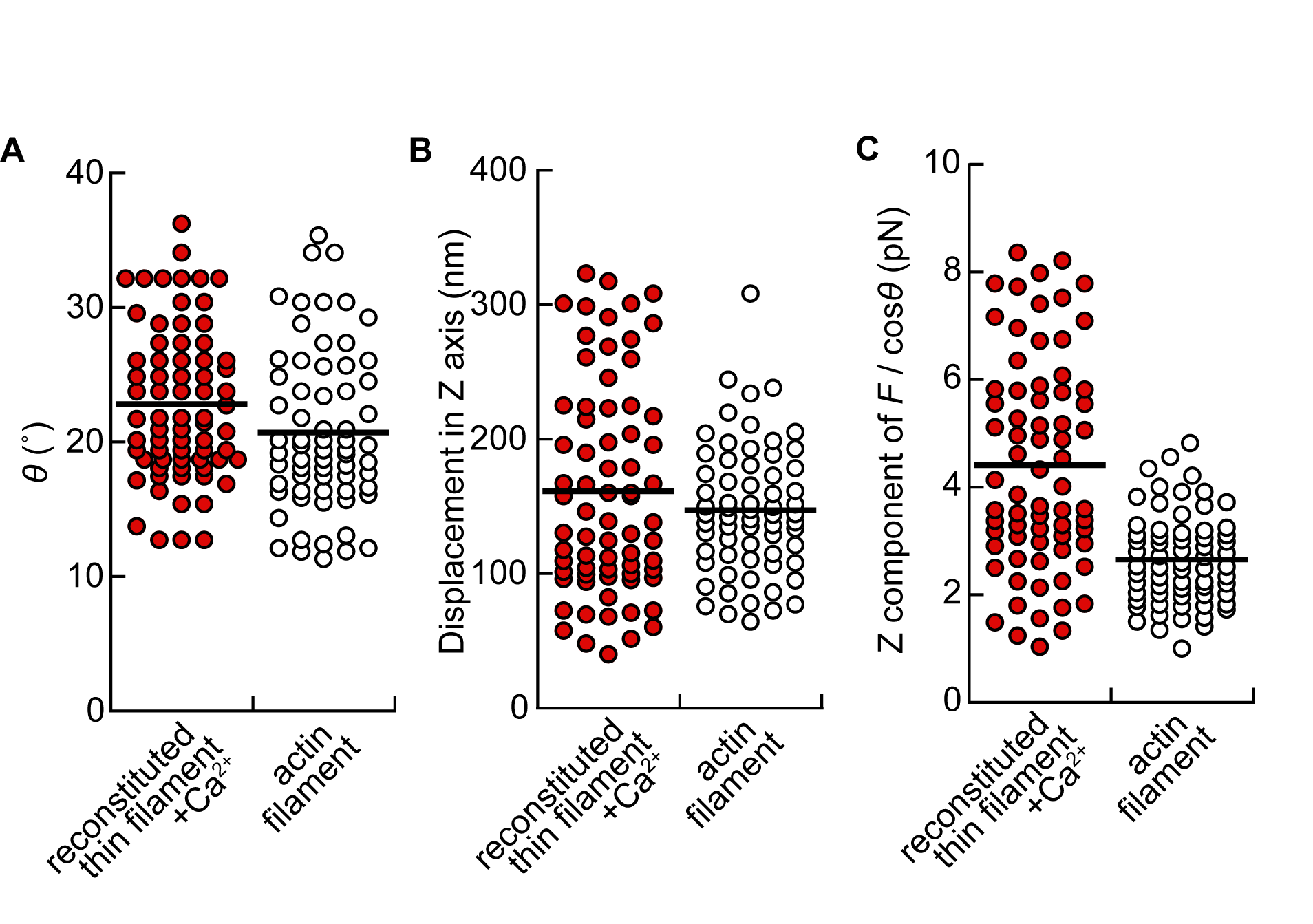

Supplement: S2 Fig — (A) Distributions of θ. Reconstituted thin filament, 23 ± 5.6°. Actin filament, 21 ± 5.9°. (B) Displacement of the bead in the Z axis calculated from F, θ and the trap stiffness in the Z axis that was assumed to be one-fifth of that in X-Y plane as previously reported [59]. Reconstituted thin filament; 161 ± 78 nm. Actin filament; 147 ± 48 nm. (C) Distribution of the perpendicular component of sliding force with a compensation of the angle of force vector (F/cosθ). Reconstituted thin filament; 4.4 ± 2.0 pN/μm. Actin filament; 2.7 ± 0.84 pN/μm. Red and white symbols represent data obtained from reconstituted thin filaments (n = 71) and actin filaments (n = 66), respectively. Horizontal bars indicate average values. (TIF) [file pone.0192558.s002.tif]

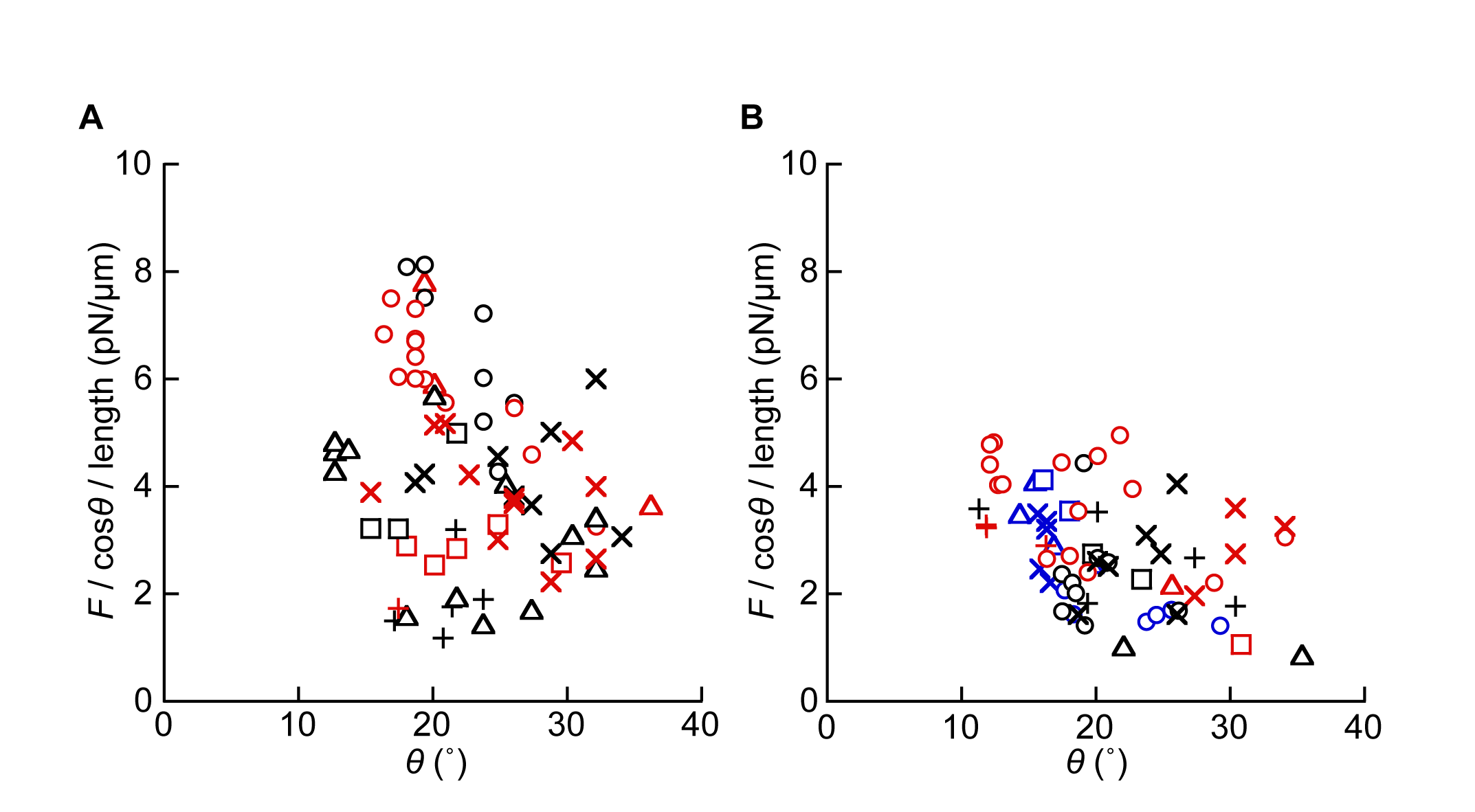

Supplement: S3 Fig — Data were reproduced from Fig 5C for reconstituted thin filaments (A) and actin filaments (B). Plots with the same style and color were obtained from the same preparation. The number of flow cells is 10 (A) and 15 (B). (TIF) [file pone.0192558.s003.tif]
